# Protocol for the preparation of zebrafish whole heart cell suspension for single-cell analyses

**DOI:** 10.1016/j.xpro.2024.103586

**Published:** 2025-01-18

**Authors:** Karim Abu Nahia, Cecilia Lanny Winata

**Affiliations:** 1 International Institute of Molecular and Cell Biology in Warsaw, 02-109 Warsaw, Poland

**Keywords:** Cell Biology, Single cell, Developmental biology, Genomics

## Abstract

Obtaining viable cell suspension that accurately represents the diversity of complex tissues is challenging due to the distinct characteristics of each cell type. Here, we present a protocol for preparing a single-cell suspension of the zebrafish embryonic whole heart, detailing steps for heart extraction, cell dissociation, quantification, and quality assessment. This suspension is compatible with downstream analysis on various single-cell platforms.

For details on the use and execution of this protocol, please refer to Abu Nahia et al.^1^

## Highlights

• Protocol for preparing single-cell suspension from zebrafish embryonic whole heart
• Heart extraction and cell dissociation steps for a homogenous, viable cell suspension
• Guide to single-cell suspension quality check
• Suitable for downstream analysis on various single-cell platforms

## Abstract (editor highlights)

Publisher’s note: Undertaking any experimental protocol requires adherence to local institutional guidelines for laboratory safety and ethics.

## Graphical abstract

**Figure:**
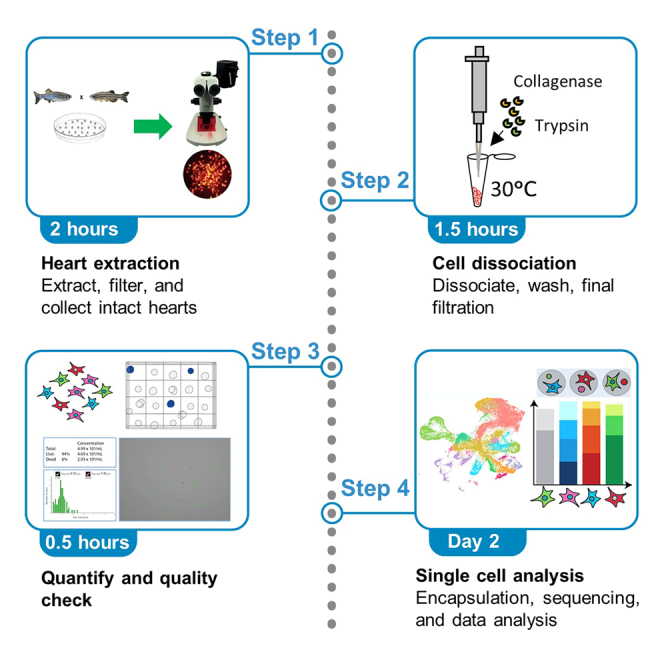

## Before you begin

**Figure 1:**
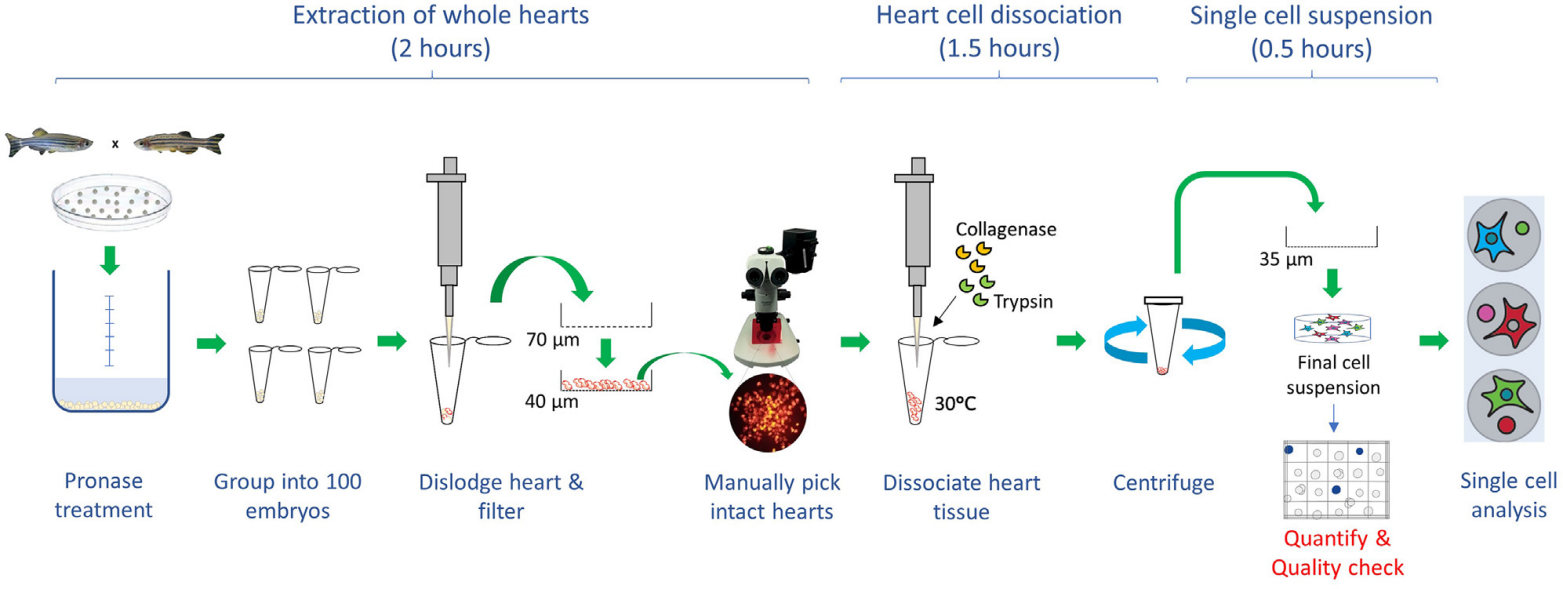
Methods workflow

The protocol below describes the specific steps for preparing homogenous and viable single cell suspension from the zebrafish whole heart for downstream single cell transcriptome analysis (Figure 1). In our previous study, we have used the 10X Genomics system; however, the cell suspension preparation should be compatible with other commercial single cell platforms with adjustments of starting cell concentrations required by the respective systems. To achieve 10,000 captured cells on the 10X Genomics platform, we typically start with 1,000 to 1,500 embryos. This protocol has been optimized for 48 and 72 h post-fertilization (hpf) zebrafish embryos. However, it should also be possible to adjust the enzymatic digestion timings for embryos of different developmental stages.

### Institutional permissions

Zebrafish wild-type and transgenic lines used in this study were maintained in the zebrafish facility of the International Institute of Molecular and Cell Biology in Warsaw (license no. PL14656251) in line with standard procedures and ethical guidelines. The experiments in this study involved only zebrafish larvae below the age of 120 hpf, which does not fall under the definition of animal experimentation according to the EU Directive 2010/63/EU on the protection of animals used for scientific purposes. Users of this protocol should acquire permission for conducting experiments on zebrafish in advance from the relevant institutions where necessary.

## Key resources table

**Table:** 

REAGENT or RESOURCE	SOURCE	IDENTIFIER
**Chemicals, peptides, and recombinant proteins**		

Tricaine	Sigma-Aldrich	A5040
Leibovitz’s L-15 medium	Gibco	21083-027 (alternative: Thermo Fisher 11415064)
10% Fetal bovine serum	Sigma-Aldrich	F2442
Collagenase	Sigma-Aldrich	C0130
0.25% Trypsin-EDTA	Corning	25-051-CI (alternative: Thermo Fisher 25200056)
Proteinase K	Roche	3115836001
Pronase from *Streptomyces griseus*	Roche	10165921001
Bovine serum albumin	Thermo Scientific	B14
Trypan blue solution (0.4%)	Thermo Scientific	15250061
“Instant Ocean” sea salt	Instant Ocean	SS6-25

**Experimental models: Organisms/strains**		

*Tg(myl7:EGFP)*	Huang et al., 2003^2^	ZFIN: ZDB-ALT-050809-20
*Tg(myl7:EGFP-Hsa.HRAS)s883*	D’Amico et al., 2007^3^	ZFIN: ZDB-ALT-070309-1
*Tg(myl7:mRFP)*	Rohr et al., 2008^4^	ZFIN: ZDB-ALT-080917-1
*sqet31Et*	Poon et al., 2010^5^	ZFIN: ZDB-ALT-070702-10
*sqet33mi59BEt*	Poon et al., 2016^6^	ZFIN: ZDB-ALT-110630-4

**Other**		

70 μm Nylon cell strainer	VWR	732–2758
40 μm Nylon cell strainer	VWR	732-2757
35 μm FACS tube cell strainer	Falcon	352235
Gel-loading pipette tips	VWR	732-3670
Cell chamber slides	NanoEntek	EVS-050
Countess 3 automated cell counter	Invitrogen	AMQAX2000
SZX16 fluorescent stereomicroscope	Olympus	SXZ16-ILLB
Axio Imager.M2	Zeiss	3525001692
Benchrocker 3D	Benchmark	B3D2300
Benchtop centrifuge	Eppendorf	5425R
DNA lo-bind microcentrifuge tubes, 2 mL	Eppendorf	0030108078
Glass Petri dish	VWR	391–0666
ThermoMixer C	Eppendorf	5382000015
Chromium Controller	10× Genomics	https://www.10xgenomics.com/instruments/chromium-controller

## Materials and equipment

**Table:** Stock salt solution

Reagent	Final concentration	Amount
“Instant Ocean” Sea Salts	40 g/L	40 g
ddH_2_O	N/A	1 L
**Total**	**N/A**	**1 L**

Store at 25°C indefinitely.

**Table:** Egg water

Reagent	Final concentration	Amount
Stock salt solution	60 μg/mL	5 mL
ddH_2_O	N/A	1 L
**Total**	**N/A**	**1 L**

Store at 25°C indefinitely.

**Table:** Pronase stock solution

Reagent	Final concentration	Amount
Pronase from *Streptomyces griseus*	20 mg/mL	1 g
ddH_2_O	N/A	50 mL
**Total**	**N/A**	**50 mL**

Store aliquots of 1 mL at −20°C to prevent repeated freeze-thaw cycles.

**Table:** EDM solution

Reagent	Final concentration	Amount
Leibovitz’s L-15 medium	N/A	27 mL
10% Fetal Bovine Serum	1%	3 mL
**Total**	**N/A**	**30 mL**

Prepare fresh for each experiment.

**Table:** Cell dissociation mix

Reagent	Final concentration	Amount
0.25% Trypsin-EDTA	N/A	920 μL
Collagenase (100 mg/mL)	8 mg/mL	80 μL
**Total**	**N/A**	**1 mL**

Prepare fresh for each experiment.

**Table:** Cell suspension buffer

Reagent	Final concentration	Amount
PBS	N/A	990 μL
BSA (4% stock, in PBS)	0.04%	10 μL
**Total**	**N/A**	**1 mL**

4% BSA in PBS can be prepared as stock and kept in 1 mL aliquots in −20°C. Cell suspension buffer should be prepared fresh for each experiment.

## Step-by-step method details

### Extraction of whole hearts

**Timing: 2 h**

This step describes the isolation of whole heart tissues from zebrafish larvae prior to tissue dissociation. An important consideration is to prevent degeneration of heart tissues by prolonged manual isolation. Hence it is preferable to process one batch at a time after Pronase digestion. The conditions of Pronase digestion and aspiration indicated in this protocol have been optimized for 48 hpf and 72 hpf embryos. These parameters should be adjusted for embryos of different developmental stages.

1. Anesthetize embryos
  a. Anesthetize embryos at desired stage by treating with Tricaine (0.16 mg/mL in egg water).
  b. Incubate on ice for 5 min.

Troubleshooting, problem 5.

2. De-chorionate embryos.
  a. Prepare 1 mg/mL Pronase solution by combining 200 μL of Pronase stock solution with 3.8 mL of egg water.
  b. In a glass beaker, pool embryos and incubate in the Pronase solution at room temperature on a nutator at 15–20 rpm until embryos are released from the chorions.
  c. Wash embryos at least three times to remove digested chorions and remaining enzyme with ice-cold egg water.
3. Group de-chorionated embryos into multiple pools of approximately 100 in low-binding 2 mL Eppendorf tubes.

***Note:*** To prevent adverse effects associated with excessive tissue degeneration, it is preferable to process one batch of 100-embryos at a time from this point.

4. Extract intact hearts.
  a. Replace egg water with 1 mL of ice-cold EDM solution.
  b. Aspirate embryos by slowly pipetting with 200 μL gel-loading tip, carefully ensuring that the first 3–5 aspirations are accompanied by some resistance, avoiding foaming.

***Note:*** For 48 hpf embryos: 6–8 times using a 200 μL pipette tips with tips cut approximately 2 mm from the end with a sterile scalpel or surgical scissors. For 72 hpf embryos: 9–12 times using a 200 μL pipette tips with tips cut approximately 2 mm from the end with a sterile scalpel or surgical scissors.

***Note:*** The aspiration step serves to dislodge intact heart from the rest of the body. Excessive aspiration may cause damage to the heart tissue or separate the atria and ventricles. Hence, visual inspection of the samples under a light microscope could also help with optimizing the conditions for this step.

5. Size filtration.
  a. Load the solution containing embryonic tissue on a 70 μm nylon cell strainer and collect pass-through solution onto a glass Petri dish.
    ***Note:*** Large debris will be retained.
  b. Filter the resulting flow through on a 40 μm nylon cell strainer and collect pass-through solution onto a glass Petri dish.
    ***Note:*** Whole hearts are retained together with tissue fragments of similar size, while smaller fragments pass through.
  c. Perform an extra wash of empty glass Petri dish with ice-cold EDM solution to collect the remaining tissue and load again on 40 μm nylon cell strainer.
    ***Note:*** As above, hearts will be retained on the strainer. *Load the solution carefully to avoid washing out the retained hearts from the strainer.*
6. Wash embryonic tissue retained on the 40 μm strainer with ice-cold EDM solution and transfer into a clean glass Petri dish.
7. Manually separate heart from remaining tissue under a stereomicroscope.

***Note:*** As the hearts are largely detached from other tissues at this stage, this could be done simply by grouping the heart together and separating them from the rest of the tissues to aid aspiration in the next step. If isolating heart from a fluorescent transgenic line such as Tg(*myl7*:EGF), hearts can be visualized by GFP fluorescence. Otherwise, hearts can also be recognized under bright field by their autonomous contraction.

8. Collect hearts by aspirating with an uncut 200 μL pipette tip into a 2 mL low binding Eppendorf tube containing 0.5 mL of EDM solution.

**CRITICAL:** Collection of hearts from each 100-embryos batch is preferably done for at most one hour to avoid excessive tissue degradation. When performing more than a single batch collection, place the earlier batches on ice while completing the rest.

Troubleshooting, problem 1.

### Heart cell dissociation

**Timing: 1.5 h**

This step outlines the method for preparing a single-cell suspension from isolated whole hearts for subsequent single-cell RNA-seq analysis. The process involves initial centrifugation to separate heart tissue, followed by a dissociation step using a Trypsin-EDTA and collagenase mix to break down the tissue into single cells. The dissociation is halted with ice-cold EDM medium, and the cell suspension is washed and filtered to remove debris. The resulting single-cell suspension is then quantified, inspected for viability, and is ready for downstream applications. This protocol ensures high cell viability and is suitable for isolating challenging cell types, such as cardiomyocytes.

9. Spin down collected hearts.
  a. Centrifuge pools of collected hearts for 15 min at 50-100 × *g*.
  b. Transfer the supernatant to another 2 mL low-binding Eppendorf tube and keep the pellet on ice.
  c. Centrifuge the supernatant at 2400 × *g* for 2 min.
  d. Collect any remaining pellet and combine it with the main pellet from the first centrifugation.
10. Dissociate heart tissue.
  a. Resuspend the pellet in 1 mL of cell dissociation mix.
  b. Incubate the mixture in a thermomixer (Eppendorf) at 30°C for 30 min with gentle pipetting 10–15 times using a 1 mL low binding pipette tip every 5 min.
  c. After 30 min, stop the dissociation reaction by adding 1 mL ice-cold EDM.

***Note:*** The conditions for dissociation have been optimized for 48 hpf and 72 hpf embryos. Adjustments, particularly in the length of enzymatic treatment, may need to be adjusted for embryos of different developmental stages.

Troubleshooting, problems 2 and 4.

11. Wash dissociated cells.
  a. Centrifuge the dissociated mixture for 5 min at 100 × *g*.
  b. Transfer the supernatant to another 2 mL low binding and centrifuge again at 850 × *g* for 1 min.
  c. Wash the pellet with 1 mL of cell suspension buffer.
  d. Centrifuge at 850 × *g* for 1 min and discard supernatant.
  e. Resuspend pellet in 200 μL of cell suspension buffer.

***Note:*** The first centrifugation at 100 × *g* is necessary to exclude the larger, undigested tissue fragments. After the last centrifugation step at 850 × *g*, the pellet contains dissociated single cells. The final resuspension volume can be adjusted according to the desired cell concentration.

12. Final filtration.
  a. Filter the washed cell suspension through a 35 μm FACS tube cell strainer to remove any remaining debris.
    i. Attach a 35 μm FACS tube cell strainer on a low-binding Eppendorf tube and perform a quick spin in the centrifuge.
  b. Gently resuspend the filtered cell suspension in 100 μL of cell suspension buffer and mix by gentle pipetting to obtain the final single-cell suspension.

***Note:*** Resuspension volume can be adjusted if more or less concentrated cell suspension is required for different single cell protocols. Remember to account for an extra 20 μL for quantification and quality assessment.

Troubleshooting, problem 3.

### Single-cell suspension quality check

**Timing: 0.5 h**

This step details the procedure for quantifying cells and assessing their viability using Trypan blue staining and automated cell counting on the Countess cell counter, which differentiates between live and dead cells based on staining. Additionally, cells are visually inspected under a microscope to confirm quality and the absence of clumps or debris, ensuring high viability for downstream applications.

13. Stain cell suspension with Trypan blue.
  a. Gently mix the cell suspension to ensure even distribution.
  b. Mix a 10 μL aliquot of cell suspension with an equal volume of Trypan blue.
  c. Immediately load 10 μL of the Trypan blue-cell mixture into each of the two measurement chambers on the cell chamber slide, enabling replicate measurements from the same sample.
14. Automated cell counting.
  a. Load the slide chambers into the Countess cell counter which will discriminate between live and dead cells based on Trypan blue staining.
  b. Perform four measurements per cell suspension:

***Note:*** Determine percentage of viability. For most downstream single cell applications, a high viability (>80%) is essential. Calculate average cell count of the four measurements and use this average to further calculate cell input for encapsulation on the Chromium Controller (10× Genomics).

15. Microscopic Inspection of single cell suspension.
  a. Load a 10 μL aliquot of cell suspension onto cell chamber slides and visually inspect under a microscope at 10-20× objective (Zeiss Axio Imager.M2).
  b. Confirm the quality of the cell suspension based on cell morphology (round-shaped, intact cells which are separate from each other indicates viable, homogenous single cell suspension) and ensure there are no large cell aggregates or other debris present.

## Expected outcomes

A key requirement to obtain high quality single cell transcriptome is a highly viable and evenly dissociated cell suspension. This protocol aims to allow the isolation and preparation of single-cell suspension from whole hearts for downstream analysis on various commercial platforms. The resulting cell suspension is anticipated to be of high viability, suitable for downstream single-cell RNA sequencing and other single-cell analysis techniques, enabling detailed investigation of cellular and molecular mechanisms in the heart.

**Figure 2:**
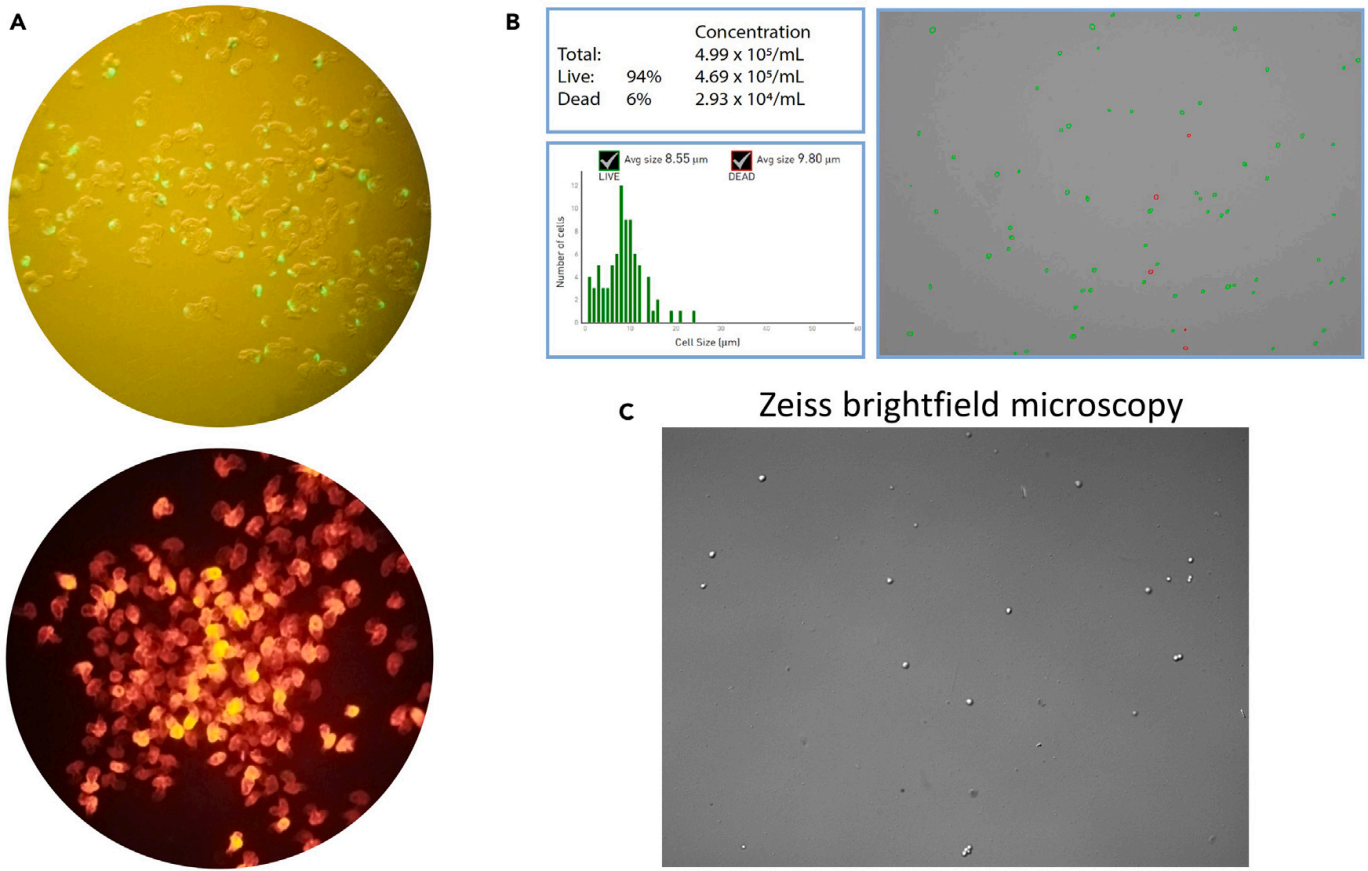
Example of the processed image showing quality and quantity check of single-cell suspension by automated cell counter and microscopy (A) Isolated hearts from *Tg(myl7:mRFP)* x *Sqet33mi59BEt* transgenic line as viewed under fluorescent microscope prior to cell dissociation. (B) Countess Automated Cell Counter showing typical yield and quality of obtained cell suspension. Viable cells are marked as green. (C) Raw images of heart dissociation metrics obtained by Countess Automated Cell Counter. To further substantiate the successful cell dissociation, each automated measurement (Countess) is supported by ZEISS brightfield microscopy (10-20× objective) taken each time before proceeding with cell encapsulation

Aiming to capture 10,000 single cells on the 10× Genomics platform, approximately 1,000 to 1,500 embryos of 48 hpf and 72 hpf stages were processed for heart isolation in each experiment. In our hands, this allowed us to reasonably obtain 400–600 intact hearts in each experiment. Depending on the sample, we typically obtain a total of 53,000–120,000 cells in a final elution volume of 100 μL. Quality check and quantification of the single cell suspension is essential before proceeding to single cell encapsulation. In our hands, this protocol typically resulted in cell suspension which is >90% in viability and evenly dissociated into individual cells without notable tissue debris (Figure 2).

One of the challenges in achieving an accurate representation of cell types in single cell analyses of heterogenous tissues is posed by the variability of the inherent characteristics of each cell type. In case of the heart, cardiomyocytes are known to be one of the most challenging cell types to isolate.^7^ Acknowledging this issue, this protocol generally allowed us to obtain a considerable proportion of cardiomyocytes from the total number of cells isolated from the whole heart, surpassing other common cardiac cell types including the mesenchyme, fibroblasts, and neural crest cells.^1^ Several studies have offered estimations of the proportion of cell types in the heart.^8^ While primarily done for the adult mammalian heart, these could still provide a tentative insight into what might be expected in the zebrafish. Although varying numbers have been suggested, the general consensus seems to be that CMs account for the second or third most abundant cell type of the heart, surpassed by endothelial cells, with the latter approximately twice as numerous. While we cannot rule out any potential bias in terms of their survivability, our dataset generally aligns with this consensus, reflecting the relative abundance of these two cell populations. Notably, in whole-embryo single cell studies, including those in the zebrafish, the depletion of CMs population is evident, and in fact much more pronounced compared to that observed in our dataset.^9^^,^^10^

By providing a robust method for isolating and analyzing heart cells, this protocol is expected to facilitate the identification of previously uncharacterized cell populations within the heart, offering new insights into cardiac development and function. The ability to capture challenging cell types like cardiomyocytes with high efficiency underscores the utility and significance of this method in cardiovascular research.

## Limitations

While this protocol allows for the isolation of cardiomyocytes up to 72 hpf, it is important to note that cardiomyocytes in the mature heart may develop distinct structural and physiological characteristics. Therefore, this protocol may not be directly applicable for isolating cells from more mature or adult hearts and would require further optimization in such case.

Additionally, the lack of reliable annotations for zebrafish cell types and their molecular markers can pose challenges in validating the comprehensive capture of cell types. However, as more single-cell transcriptome data become available for cross-referencing, this issue is expected to be progressively alleviated.

While the protocol is suitable for single-cell analysis platforms, compatibility with other downstream applications, such as cell culture or certain functional assays, may need to be verified and optimized.

## Troubleshooting

### Problem 1 (step 8)

Low cell viability and/or mechanical damage to cells.

### Potential solution

• Prolonged handling and delays between steps can lead to decreased cell viability. It is essential to minimize the time cells spend outside of controlled environments.
• Use low-binding pipette tips and gentle pipetting techniques.
• Calibrate centrifuges to ensure proper speed and avoid excessive force.
• Use a thermomixer with precise temperature control during enzymatic digestion.

### Problem 2 (step 10)

High number of cell multiplets due to incomplete dissociation.

### Potential solution

• Although the enzymatic concentrations and timings of digestion are stated in this protocol, it is crucial to optimize these parameters when running the experiment for the first time due to the potential differences in the activity of enzymes from different production batches.
• The quality and freshness of reagents, such as Trypsin-EDTA and collagenase, are critical. Degraded or expired reagents can significantly affect the efficiency of cell dissociation and viability.
• Maintaining consistent ambient temperatures throughout the protocol is crucial. Variations can affect enzyme activity during digestion and cell viability during isolation and storage.

### Problem 3 (step 12)

Excessive debris present in cell suspension.

### Potential solution

• The consistency and quality of nylon cell strainers can affect the retention and loss of cells. Poor-quality strainers may result in the loss of smaller cell types or retention of unwanted debris.
• Additionally, pre-filtering of cell media and buffers may help to exclude any molecular aggregates or precipitates that may forms during storage.

### Problem 4 (step 10)

Variability in cell type representation.

### Potential solution

Adjust the protocol to optimize conditions for the isolation of less represented cell types. Validate the protocol using known markers for different cell types to ensure comprehensive capture.

### Problem 5 (step 1)

Although rare, we occasionally encounter contamination of transcriptomic data with bacterial DNA reads from the environment. This issue is particularly pronounced when working with low input samples.

### Potential solution

Use aseptic techniques throughout the protocol. Regularly sterilize work surfaces and equipment. Use sterile, filtered reagents and consumables. Adding methylene blue to the embryo medium could also help in preventing excessive bacterial or fungal growth.

## Resource availability

### Lead contact

Further information and requests for resources and reagents should be directed to and will be fulfilled by the lead contact, Cecilia Lanny Winata (cwinata@iimcb.gov.pl).

### Technical contact

Technical questions on executing this protocol should be directed to and will be answered by the technical contact, Karim Abu Nahia (karim.abunahia@gmail.com).

### Materials availability

This study did not generate new unique reagents.

### Data and code availability

The published article includes all datasets and code generated or analyzed during this study.

## Acknowledgments

The project POIR.04.04.00-00-1AF0/16-00 is carried out within the First TEAM program of the Foundation for Polish Science co-financed by the European Union under the 10.13039/501100008530European Regional Development Fund. This research was supported by National Science Center Poland, grant number 2019/35/B/NZ2/02548. All transgenic zebrafish lines used in this study were maintained at the IIMCB zebrafish core facility (IN-MOL-CELL Infrastructure) funded by the European Union – NextGenerationEU under National Recovery and Resilience Plan. IN-MOL-CELL Infrastructure was also funded by the European Union under 10.13039/100018693Horizon Europe (project 101059801 - RACE) and RACE-PRIME project carried out within the IRAP programme of the Foundation for Polish Science co-financed by the European Union under the European Funds for Smart Economy 2021–2027 (FENG).

## Author contributions

C.L.W. conceived the project and designed the experiments together with K.A.N. K.A.N. performed the experiments and analysis. C.L.W. wrote the manuscript with input from K.A.N. All authors have read and approved the final paper.

## Declaration of interests

The authors declare no competing interests.
